# Apamin structure and pharmacology revisited

**DOI:** 10.3389/fphar.2022.977440

**Published:** 2022-09-16

**Authors:** Alexey I. Kuzmenkov, Steve Peigneur, Joshua A. Nasburg, Konstantin S. Mineev, Maxim V. Nikolaev, Ernesto Lopes Pinheiro-Junior, Alexander S. Arseniev, Heike Wulff, Jan Tytgat, Alexander A. Vassilevski

**Affiliations:** ^1^ Shemyakin-Ovchinnikov Institute of Bioorganic Chemistry, Russian Academy of Sciences, Moscow, Russia; ^2^ Toxicology and Pharmacology, KU Leuven, Leuven, Belgium; ^3^ Department of Pharmacology, University of California, Davis, Davis, CA, United States; ^4^ Moscow Institute of Physics and Technology, Moscow Region, Dolgoprudny, Russia; ^5^ Sechenov Institute of Evolutionary Physiology and Biochemistry, Russian Academy of Sciences, Saint Petersburg, Russia

**Keywords:** apamin, *Apis mellifera*, bee venom, calcium-activated potassium channel, ion channel, spatial structure

## Abstract

Apamin is often cited as one of the few substances selectively acting on small-conductance Ca^2+^-activated potassium channels (K_Ca_2). However, published pharmacological and structural data remain controversial. Here, we investigated the molecular pharmacology of apamin by two-electrode voltage-clamp in *Xenopus laevis* oocytes and patch-clamp in HEK293, COS7, and CHO cells expressing the studied ion channels, as well as in isolated rat brain neurons. The microtitre broth dilution method was used for antimicrobial activity screening. The spatial structure of apamin in aqueous solution was determined by NMR spectroscopy. We tested apamin against 42 ion channels (K_Ca_, K_V_, Na_V_, nAChR, ASIC, and others) and confirmed its unique selectivity to K_Ca_2 channels. No antimicrobial activity was detected for apamin against Gram-positive or Gram-negative bacteria. The NMR solution structure of apamin was deposited in the Protein Data Bank. The results presented here demonstrate that apamin is a selective nanomolar or even subnanomolar-affinity K_Ca_2 inhibitor with no significant effects on other molecular targets. The spatial structure as well as ample functional data provided here support the use of apamin as a K_Ca_2-selective pharmacological tool and as a template for drug design.

## 1 Introduction

Highly selective molecules that can interact with specific ion channel isoforms serve as invaluable molecular tools for fundamental and applied pharmacology (Hille, 2001; Wulff et al., 2019). The history of using such substances goes hand-in-hand with the discovery and investigation of their molecular targets (Hille, 2001). Indeed, selective molecular probes helped to identify and characterize a number of physiologically critical ion channels (Kuzmenkov and Vassilevski, 2018). For example, marine guanidinium and scorpion polypeptide toxins were used in pioneering studies for the purification of voltage-gated Na^+^ channels (Na_V_) (Hartshorne and Catterall, 1981; Barhanin et al., 1983; Hanke et al., 1984). Moreover, Na_V_ are traditionally classified based on isoform sensitivity to tetrodotoxin (Narahashi, 2008). A plethora of other ion channel-selective compounds has been extracted from different animal venoms (Herzig et al., 2020).

In the 1960s, Habermann examined the venom of the honeybee *Apis mellifera* and purified a minor peptide component that he named “apamin” (Habermann and Reiz, 1965; Habermann, 1972); the toxin was shown to induce muscle spasms, jerks, and convulsions when injected into mice (LD_50_ ≈ 4 mg kg^−1^, i.v.). The symptoms are apparently of central origin because the toxicity increases by a factor of 1,000–10,000 and the progression of poisoning is faster in case of i.c.v. injection, but the clinical picture stays the same (Habermann, 1977, Habermann, 1984). Primary structure determination showed that apamin contains 18 amino acid residues (Haux et al., 1967; Shipolini et al., 1967), with two intramolecular disulfide bonds (Cys1–Cys11 and Cys3–Cys15) (Callewaert et al., 1968). Moreover, the C-terminal residue is amidated; this post-translational modification is common for peptide toxins from animal venoms (Kuzmenkov et al., 2015).

Studies of the spatial structure of apamin began as early as the late 1970s, but no atomic coordinates have been deposited in the Protein Data Bank. The first NMR study provided an erroneous assignment of the secondary structure elements (Bystrov et al., 1980), which was corrected in further works (Wemmer and Kallenbach, 1983; Pease and Wemmer, 1988). The most recent work (Le-Nguyen et al., 2007) presented both a solution NMR structure of apamin and an X-ray structure of its N-terminally acetylated analog, which differ in the packing of the N-terminal region, the length of the ɑ-helix, and the χ^3^ angles of the disulfide bridges. In addition, whereas apamin is monomeric in solution, the X-ray structure is a dimer, and that dimeric structure was deposited in the Cambridge Crystallographic Data Centre (https://www.ccdc.cam.ac.uk; database identifier: NIWFEF).

Apamin played a key role in the identification and characterization of a novel subset of Ca^2+^-activated K^+^ ion channels (K_Ca_) (Banks et al., 1979; Lazdunski, 1983; Garcia et al., 1991). Since the early 1980s, this toxin has been widely utilized to differentiate K_Ca_ channels into “apamin-sensitive” and “apamin-insensitive” because it selectively affected only the small-conductance K_Ca_ channels (K_Ca_2, SK2, or SK_Ca_) and not intermediate-conductance (K_Ca_3.1) or large-conductance (K_Ca_1.1) K_Ca_ channels. (Burgess et al., 1981; Romey and Lazdunski, 1984; Pennefather et al., 1985). After the cloning of SK channels from rat and human brain and their expression in *Xenopus laevis* oocytes, the direct activity of apamin was shown on these molecular targets (Köhler et al., 1996; Grunnet et al., 2001a). A number of pharmacological studies using *X*. *laevis* oocytes and mammalian cells indicated that among the three SK isoforms apamin was more potent on K_Ca_2.2 (SK2 or SK_Ca_2; IC_50_ = 0.03–0.14 nM) and less potent on K_Ca_2.1 (SK1 or SK_Ca_1; IC_50_ = 0.7–12 nM) (Köhler et al., 1996; Shah and Haylett, 2000; Strøbaek et al., 2000; Grunnet et al., 2001a), whereas K_Ca_2.3 (SK3 or SK_Ca_3) channels showed an intermediate sensitivity (IC_50_ = 0.6–4.0 nM) (Ishii et al., 1997; Grunnet et al., 2001b; Grunnet et al., 2001a).

Those investigations led to a prevailing view in the literature that apamin is a selective probe for just SK channels. Quite surprisingly, data to support this claim by showing absence of activity on other targets are missing. Moreover, some published studies actually claim various off-target activities of apamin. For example, it was assumed that apamin affects Ca^2+^ and/or Na^+^ channels in the embryonic chicken heart (Bkaily et al., 1985; Bkaily et al., 1991; Bkaily et al., 1992). On the contrary, in another study human cardiac Ca^2+^, Na^+^, or K^+^ channels were not affected, with the exception of SK channels (Yu et al., 2014). Apamin was also reported to affect inward-rectifier K^+^ channels K_ir_3.1/3.4 and the voltage-gated K^+^ channel K_V_1.3 (Jin and Lu, 1998; Voos et al., 2017); however, the activity of the toxin on those channel isoforms expressed individually is yet to be shown.

To verify the molecular pharmacology of such an important compound as apamin and to resolve the contradictory claims, we performed a large-scale electrophysiological profiling of this toxin against various molecular targets. We conclude that apamin is indeed a selective inhibitor of small-conductance K_Ca_ channels.

## 2 Materials and methods

### 2.1 Materials

For consistency, we used apamin purified from the honeybee *Apis mellifera* venom (product number A1289) and synthetic melittin (M4171) purchased from Sigma-Aldrich. TRAM-34 was synthesized as described (Wulff et al., 2000). Other low-molecular-weight compounds were purchased from Sigma-Aldrich: acetylcholine chloride (ACh; A6625), capsaicin (M2028), capsazepine (C191), glycine (G7126), kainic acid monohydrate (K0250), and N-methyl-D-aspartic acid (NMDA; M3262).

### 2.2 Nomenclature of targets and ligands

Ion channel targets and their ligands are presented according to IUPHAR/BPS Guide to PHARMACOLOGY (http://www.guidetopharmacology.org) and are permanently archived in the Concise Guide to PHARMACOLOGY 2021/22 (Alexander et al., 2021). For potassium channel ligands of protein and peptide nature, readers are advised to consult the Kalium database (https://kaliumdb.org) (Kuzmenkov et al., 2016; Tabakmakher et al., 2019).

### 2.3 Analytical chromatography

Apamin purity was confirmed using reversed-phase (RP) HPLC on a Vydac 218TP54 C_18_ column (4.6 × 250 mm; Separations Group) in a linear gradient of acetonitrile concentration (0%–60% in 60 min) in the presence of 0.1% trifluoroacetic acid (TFA). 1525 Binary HPLC pump and 2489 UV/Visible detector under the control of Breeze 2 software (all from Waters) were used for chromatography.

### 2.4 Mass spectrometry

Molecular mass measurements were performed using MALDI on an Ultraflex III TOF-TOF instrument (Bruker). 2,5-Dihydroxybenzoic acid (Sigma-Aldrich) was used as a matrix. Measurements were carried out in the reflector mode, which enabled isotopic resolution. Calibration was performed using the ProteoMass Peptide MALDI-MS Calibration Kit (Sigma-Aldrich). Mass spectra were analyzed with the Data Analysis 4.3 and Data Analysis Viewer 4.3 software (Bruker).

### 2.5 Peptide concentration measurements

Apamin and melittin concentrations were determined by UV spectrophotometry. To obtain absorption spectra of those substances in the UV range, lyophilized peptides were dissolved in 0.5 ml of water (Milli-Q produced on a Merck Millipore Water Purification System). We used a UV-1800 spectrophotometer (Shimadzu) and quartz cuvettes with an optical path length of 1.0 cm; water served as a reference solution. Apamin does not contain aromatic amino acid residues; therefore, absorbance at 205 nm was used to measure the concentration (Anthis and Clore, 2013). Melittin contains tryptophan, and so its absorbance was measured at 280 nm and the calculations were performed accordingly (Beaven and Holiday, 1952).

### 2.6 Antimicrobial assay

Apamin was tested against Gram-positive (*Enterococcus faecalis* ATCC 29212, *Staphylococcus aureus* subsp. *aureus* ATCC 29213) and Gram-negative bacteria (*Escherichia coli* ATCC 25922, *Pseudomonas aeruginosa* ATCC 27853) following the previously described modification of the microtitre broth dilution method (Vassilevski et al., 2010). Those bacterial strains were also subjected to the treatment by melittin in the same concentration range to serve as a positive control.

Briefly, bacteria were cultured in a low-salt LB medium. The two-fold microtitre broth dilution assay was carried out in 96-well sterile plates in a final volume of 100 μL. Mid-exponential-phase cultures were diluted to a final concentration of 10^5^ colony-forming units·ml^−1^. Pure peptides were dissolved in 10 μL of water and added to 90 μL of the bacterial dilution. The samples, a non-treated control and a sterility control were tested in five independent experiments (*n* = 5). The microtitre plates were incubated for 24 h at 37°C, and growth inhibition was determined by measuring the absorbance at 620 nm. Minimum inhibitory concentration (MIC) is expressed as the lowest concentration of peptide that caused 100% bacterial growth inhibition.

### 2.7 NMR spectroscopy

For NMR studies 1 mg of apamin was dissolved in 320 μL of H_2_O/D_2_O mixture (95:5) and placed into a 5-mm Shigemi NMR tube. The sample pH was adjusted to 3.2. NMR spectra of apamin were recorded using a 600-MHz Bruker Avance III NMR spectrometer, equipped with a triple-resonance cryogenic probe, at 25°C. We recorded the following spectra: DQF-COSY, NOESY (120 ms), ROESY (200 ms), TOCSY (80 ms), ^13^C and ^15^N-HSQC at natural abundance. ^3^J_HNHA_ were measured by the line shape analysis of cross-peaks in NOESY spectra, whereas ^3^J_HAHB_ couplings of AMX spin systems were determined by the line shape analysis of cross-peaks in 2D TOCSY spectra.

3D structure calculation was performed using the simulated annealing/molecular dynamics protocol as implemented in the CYANA software package version 3.98 (Güntert et al., 1997). The disulfide linkages were introduced based on previously published data (Callewaert et al., 1968). 100 structures were obtained starting from random conformations and the 10 best were then selected for further analysis. Visual inspection of the calculated structures and figure drawings were performed using PyMOL (Schrödinger) and MOLMOL (Koradi et al., 1996) software.

### 2.8 Expression of ion channels in *X. laevis* oocytes

The following genes encoding ion channel subunits were expressed in *Xenopus* oocytes: for voltage-gated potassium channels, K_V_ [rK_V_1.1 (GenBank accession number: NM_173095), rK_V_1.2 (NM_012970), hK_V_1.3 (NM_002232), rK_V_1.4 (NM_012971), rK_V_1.5 (NM_012972), rK_V_1.6 (NM_023954), hK_V_2.1 (NM_004975), hK_V_3.1 (NM_004976), rK_V_4.3 (NM_031739), hK_V_7.1 (NM_000218), hK_V_7.2/7.3 (NM_004518/NM_004519), hK_V_10.1 (EAG1; NM_172362), hK_V_11.1 (hERG; NM_000238), *Shaker*-IR from *Drosophila melanogaster* (NM_167595; amino acids 6–46 deleted), and KQT-1 from *Caenorhabditis elegans* (NM_171710)], inward-rectifier potassium channels, K_ir_ [mK_ir_3.1/3.2 or GIRK1/2 (NM_008426/XM_011246104)], voltage-gated sodium channels, Na_V_ [rNa_V_1.1 (NM_030875), rNa_V_1.2 (NM_012647), rNa_V_1.3 (NM_013119), rNa_V_1.4 (NM_013178), hNa_V_1.5 (NM_198056), mNa_V_1.6 (NM_001077499), hNa_V_1.7 (NM_002977), rNa_V_1.8 (NM_017247), rβ1 (NM_001271045), hβ1 (NM_001037), and the arthropod channels BgNa_V_1 from *Blattella germanica* (U73583) and VdNa_V_1 from *Varroa destructor* (AY259834), and TipE from *D. melanogaster* (NM_079196)], transient receptor potential channels, TRP (human TRPV1, NM_080704), nicotinic acetylcholine receptors, nAChR [human α1β1γδ (NM_001039523, NM_000747, NM_005199, NM_000751), α4β2 (NM_000744, NM_000748), and α7 (NM_000746)].

Linearized plasmids bearing the ion channel genes were transcribed using the mMESSAGE mMACHINE SP6 or T7 transcription kits (Ambion) to prepare the respective cRNA. The harvesting of stage V–VI oocytes from anaesthetized female *X. laevis* frogs was described previously (Liman et al., 1992; Peigneur et al., 2021). Oocytes were injected with 50 nL of cRNA at a concentration of 1 ng nl^−1^ using a microinjector (Drummond Scientific). The oocytes were incubated at 16°C in ND96 solution containing (in mM): NaCl, 96; KCl, 2; CaCl_2_, 1.8; MgCl_2_, 2; and HEPES, 5 (pH 7.4), supplemented with 50 mg l^−1^ gentamicin sulfate.

### 2.9 Expression of ion channels in eukaryotic cells

HEK293 cells stably expressing hK_Ca_1.1 (BK, GenBank accession number: NM_002247), hK_Ca_2.1 (SK1, NM_002248), and hK_Ca_3.1 (IK, AH009923) or COS7 cells stably expressing hK_Ca_2.3 (SK3, AJ251016) were generated and cultured as previously described (Sankaranarayanan et al., 2009). HEK293 cells stably expressing rK_Ca_2.2 (SK2, NM_019314) were a gift from Dr. Mio Zhang (Chapman University, Irvine).

CHO cells were used for transient expression of acid-sensing ion channels (ASIC). These cells were cultured in a CB-150 CO_2_ incubator (Binder) at 37°C in a humidified atmosphere of 5% CO_2_. Cells were maintained under standard culture conditions (DMEM/F12, 10% fetal bovine serum, and 50 mg l^–1^ gentamicin) in 35 mm^2^ Petri dishes. Transfection was performed using 0.5 μg of plasmids encoding rASIC1a (NM_024154), rASIC2a (NM_001034014), or rASIC3 (NM_173135) gifted by Dr. Alexander Staruschenko (University of South Florida, Tampa) with 0.5 μg of a GFP-encoding plasmid and Lipofectamine 2000 (Invitrogen) according the manufacturer’s protocol. Patch-clamp experiments were performed 48 h after transfection.

### 2.10 Isolation of rat neurons

Wistar rats (12–18 days old, both sexes) were deeply anaesthetized with isoflurane and sacrificed by cervical dislocation followed by decapitation. The brains were quickly removed and immersed in ice-cold (2°C–4°C) artificial cerebrospinal fluid (ACSF) of the following composition (in mM): NaCl, 124; KCl, 5; CaCl_2_, 1.3; MgCl_2_, 2; NaHCO_3_, 26; NaH_2_PO_4_, 1.24; and D-glucose, 10; aerated with carbogen (95% O_2_, 5% CO_2_). Transverse slices, comprising hippocampus and striatum, were cut with a 7000 SMZ-2 vibratome (Campden Instruments) and stored at room temperature (22°C–24°C) in ACSF aerated with carbogen. Neurons were isolated from the slices by vibrodissociation (Vorobjev, 1991). The effects of apamin on ionotropic glutamate receptors (iGluR) were studied on hippocampal pyramidal neurons of the CA1 area expressing GluN2A/B NMDA receptors (Monyer et al., 1994; Foster et al., 2010) and Ca^2+^-impermeable GluA2-containing AMPA receptors (Wenthold et al., 1996; Seifert et al., 2000) and on giant cholinergic interneurons of the striatum expressing Ca^2+^-permeable GluA2-lacking AMPA receptors (Buldakova et al., 1999). The experiments on ASIC were carried out on hippocampal interneurons of the *lacunosum-moleculare* and *radiatum* layers of the CA1 region, which express ASIC1a/2 heteromers (Weng et al., 2010).

### 2.11 Two-electrode voltage-clamp

Recordings were performed at room temperature (18°C–22°C) using a Geneclamp 500 amplifier (Molecular Devices) controlled by a pClamp data acquisition system (Axon Instruments). Whole-cell currents from oocytes were recorded 1–4 days after cRNA injection. Bath solution composition was ND96, or HK containing (in mM): NaCl, 2; KCl, 96; CaCl_2_, 1.8; MgCl_2_, 2; and HEPES, 5 (pH 7.4). Voltage and current electrodes were filled with 3 M KCl. Resistances of both electrodes were kept at 0.7–1.5 MΩ. Elicited currents were sampled at 1 kHz and filtered at 0.5 kHz (for potassium currents) or sampled at 20 kHz and filtered at 2 kHz (for sodium currents) using a four-pole low-pass Bessel filter. Leak subtraction was performed using a −P/4 protocol.

Currents were evoked by a 100 ms (Na_V_) or 500 ms (K_V_) depolarization to the voltage corresponding to the maximal activation of the channels in control conditions from a holding potential of −90 mV. For nAChR experiments, the oocytes were voltage-clamped at a holding potential of −70 mV and continuously superfused with ND96 via gravity-fed tubes at 0.1–0.2 ml min^−1^, with 5 min incubation times for the bath-applied peptides. ACh was applied via gravity-fed tubes until peak current amplitude was obtained (1–3 s), with 1–2 min washout periods between applications. The nAChR were gated by a particular time duration pulse of ACh for the respective nAChR subtype (200 μM for α1β1γδ and α4β2; 100 μM for α7) at 2 ml min^−1^. Data were sampled at 500 Hz and filtered at 200 Hz. TRP currents were measured in ND96 at −90 mV during 400 s. Capsaicin (2 μM) was used as an agonist and capsazepine (10 μM) as an antagonist of TRPV1. Peak current amplitude was measured prior to and following the application of the peptide. All data were obtained in at least five independent experiments (*n* ≥ 5).

### 2.12 Patch-clamp of HEK293 or COS7 cells

All experiments were conducted with an EPC-10 amplifier (HEKA) in the whole-cell configuration with a holding potential of –80 mV. Pipette resistances averaged around 2.5 MΩ. Solutions of apamin in Na^+^ Ringer were freshly prepared during the experiments from 100 μM stock solutions in Roswell Park Memorial Institute medium (RPMI). For current measurements, we used an internal pipette solution containing (in mM): K^+^ aspartate, 160; MgCl_2_, 2.08; HEPES, 10; EGTA, 10; and CaCl_2_, 8.55 (1 μM free Ca^2+^); pH 7.2; osmolarity 310 mOsm. Free Ca^2+^ concentrations were calculated with MaxChelator (developed by Chris Patton, Stanford University) assuming a temperature of 25°C, pH 7.2, and ionic strength of 160 mM. Na^+^ Ringer was used as an external solution containing (in mM): NaCl, 160; KCl, 4.5; CaCl_2_, 2; MgCl_2_, 1; and HEPES, 10; pH 7.4; osmolarity, 315 mOsm. Please note that the provided concentrations are what was weighed in to achieve an initially hyperosmolar solution that was diluted with water to 310 mOsm for the internal and 315 mOsm for the external solution as measured with a VAPRO Vapor pressure osmometer (Wescor). A slight 5 mOsm difference in osmolarity between internal and external solution improves the sealing rate.

K_Ca_2 and K_Ca_3.1 currents were elicited by 200-ms voltage ramps from –120 to 40 mV applied every 10 s, and the fold decrease of slope conductance at –80 mV was taken as a measure of apamin-induced channel inhibition. TRAM-34 was used to block K_Ca_3.1. K_Ca_1.1 channel activity was recorded with a step protocol, in which cells were stepped to +40 mV for 100 ms at 30-s intervals. Concentration-dependent current inhibition was fitted with the Hill equation using Prism 8 (GraphPad Software). All data were collected in at least five independent experiments (*n* ≥ 5).

### 2.13 Patch-clamp of rat neurons or CHO cells

In this case, the holding potential was set at –70 mV. Signals were filtered at 10 kHz and sampled at 20 kHz. Drugs were applied using an RSC-200 (BioLogic) perfusion system. The extracellular solution contained (in mM): NaCl, 143; KCl, 5; CaCl_2_, 2.5; D-glucose, 10; and HEPES, 10; and the pH was adjusted to 7.4 with HCl. 10 mM MES was used in the extracellular solution in experiments on ASIC. The patch pipettes (2.5–3.5 MΩ) were made from borosilicate glass (WPI) using a P-97 puller (Sutter Instruments). The pipette solution contained (in mM): CsF, 100; CsCl, 40; NaCl, 5; CaCl_2_, 0.5; EGTA, 5; and HEPES, 10; and the pH was adjusted to 7.2 with CsOH. All experiments were performed at room temperature (22°C–24°C). NMDA receptors were activated by 100 μM NMDA and 10 μM glycine. AMPA receptors were activated by 100 μM kainate. ASIC of rat neurons were activated by pH drops from pH 7.4 to 6.5. ASIC1a, ASIC2a, and ASIC3 homomers were expressed in CHO cells and activated by pH 6.5, 5.0, and 6.8, respectively. Lyophilized apamin was freshly dissolved in extracellular solution prior to the experiment. After recording the control response, the patched cell was superfused for 40 s with extracellular solution containing apamin, and then the response to co-application of the agonist and apamin was recorded. Offline data analyses were performed using Origin 9.1 (OriginLab) software. All data were collected from at least five experiments (*n* ≥ 5).

### 2.14 Data analysis and statistics

Data and statistical analysis comply with recommendations on experimental design and analysis in pharmacology (Curtis et al., 2018). All data points in the apamin concentration-response curves on K_Ca_ channels are means ± standard deviation (SD) from at least five independent experiments. IC_50_s are reported with 95% confidence intervals (CI). The CI is based on the fit for the averaged currents. Student’s two-tailed unpaired *t*-test was used to assess the significance of the effect of apamin in rat neurons (current in the presence of apamin vs current in control). A value of *p* < 0.05 was considered statistically significant.

## 3 Results

### 3.1 Confirmation of apamin sample purity

**FIGURE 1:**
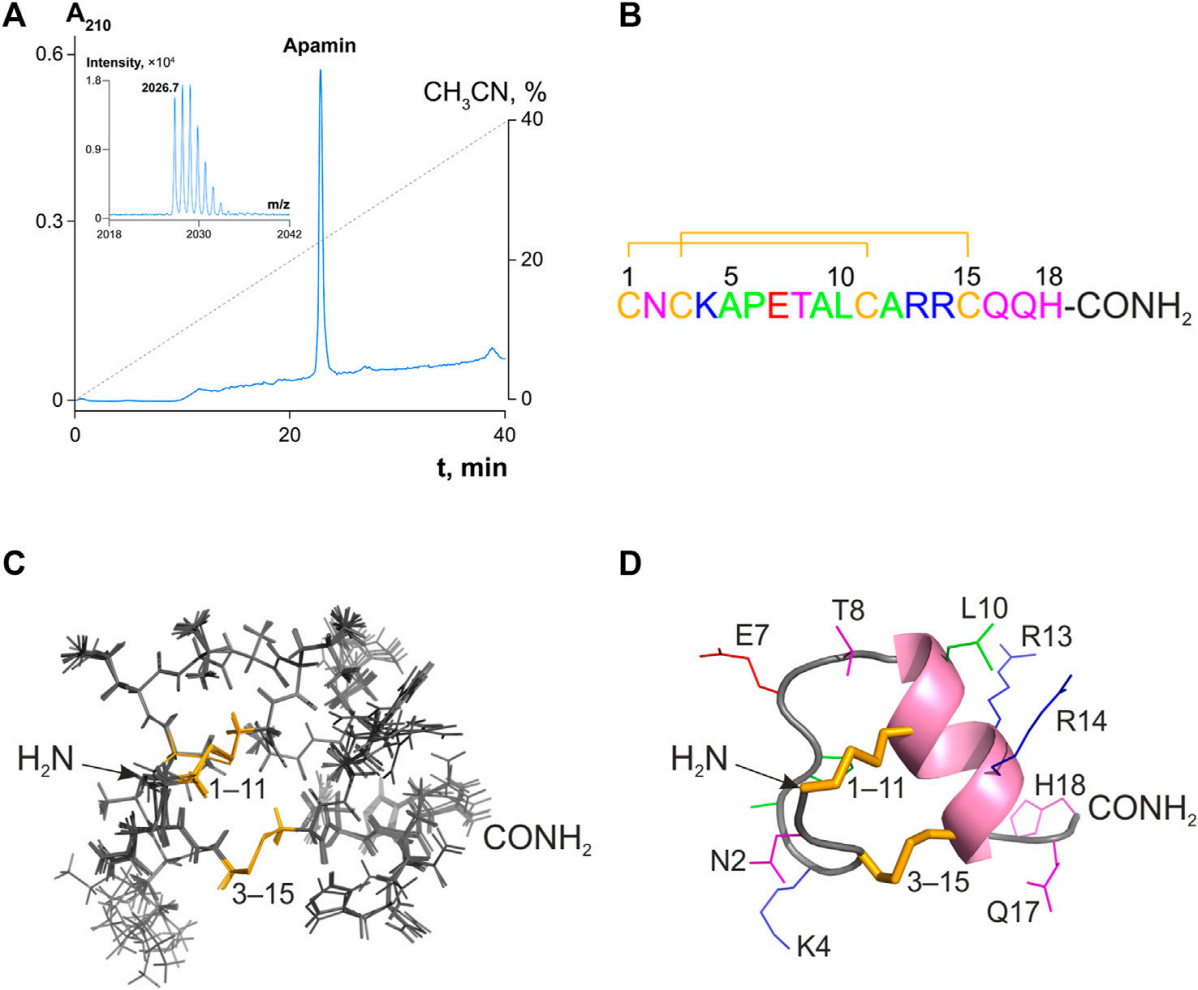
Analytical RP-HPLC of apamin and its structure in solution. **(A)** 10 nmol of peptide was injected onto a Vydac C_18_ column (4.6 × 250 mm). Reflector-mode MALDI mass spectrum with an isotopic resolution of the purified peptide is shown in the inset. **(B)** Amino acid sequence of apamin. The numbering is shown above. Amino acid residues are colored with respect to their chemical properties: positively charged residues are shown in blue, negatively charged in red, hydrophobic in green, hydrophilic uncharged in magenta, and cysteines in yellow. Disulfide bridges are shown with lines. **(C)** A set of 10 NMR structures with the fewest restraint violations (PDB ID: 7OXF). Disulfide bridges are colored in yellow and labeled. **(D)** The spatial structure of apamin is shown in a ribbon representation. The ɑ-helix is colored pink, and the rest of the main chain is gray. Side chains of the peptide are labeled and colored, the coloring is as in panel **(B)**. Disulfide bridges are shown as yellow sticks.

Natural apamin isolated from bee venom was purchased from Sigma and subjected to analytical RP-HPLC. It presented a single symmetrical Gaussian peak (Figure 1A) corresponding to >95% purity. The fraction corresponding to the individual component was further inspected by MALDI mass spectrometry. The measured monoisotopic molecular mass ([M + H]^+^ = 2026.7 Da; Figure 1A, inset) did not differ significantly from the calculated value for apamin (2026.9 Da; *Δ* = 0.2 Da).

### 3.2 Apamin spatial structure in solution

As there is no apamin structure in the PDB, we solved it with NMR spectroscopy in solution based on the recorded J-couplings and NOE distances (Supplementary Figure S1). NMR chemical shifts, experimental restraints, and the spatial structure were deposited to the BMRB and PDB databases under the accession codes 34641 and 7OXF, respectively. The obtained structure fits all the experimental data and is characterized by a low backbone RMSD value (0.24 Å), which confirms that it is well converged (Supplementary Table S1).

Apamin forms a short ɑ-helix (Ala9–Gln16) and a β-turn (Asn2–Ala5; Figures 1B–D). Additionally, the amide group of Lys4 forms a hydrogen bond with the side chain carbonyl of Asn2, which is in agreement with the previously published decelerated hydrogen-deuterium exchange of Lys4 amide (Bystrov et al., 1980). Analysis of the structure reveals no electrostatic, π-cation, or stacking interactions that could stabilize the observed apamin conformation.

### 3.3 Antimicrobial activity

The antibacterial assay showed no activity of apamin against either Gram-positive or Gram-negative bacteria up to a concentration of 50 μM. Melittin, on the other hand, displayed the expected antimicrobial effect. Its MIC values on *E*. *faecalis*, *S*. *aureus*, *E*. *coli*, and *P*. *aeruginosa* were 0.5, 3, 1.5, and 6 μM, respectively.

### 3.4 Apamin pharmacology

**FIGURE 2:**
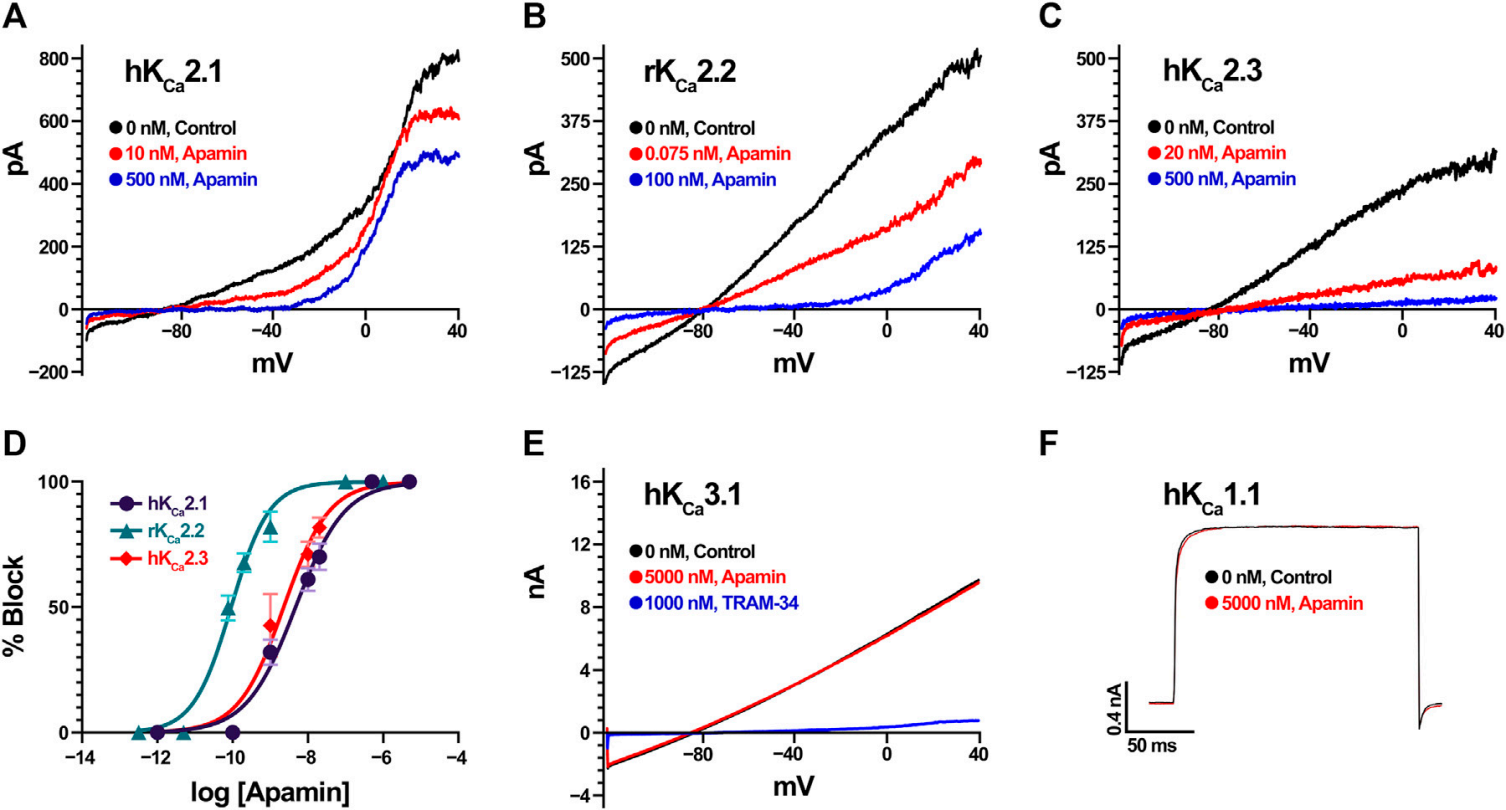
Apamin effects on K_Ca_ channels expressed in HEK293 or COS7 cells (in the case of K_Ca_2.3). **(A)** Effect of increasing concentrations of apamin (10 nM and 500 nM in red and blue, respectively) on K_Ca_2.1 currents. Note that the currents visible above 0 mV are carried by endogenous K_V_ channels in HEK cells. **(B)** Effect of increasing concentrations of apamin (75 pM and 100 nM in red and blue, respectively) on K_Ca_2.2 currents. **(C)** Effect of increasing concentrations of apamin (20 nM and 500 nM in red and blue, respectively) on K_Ca_2.3 currents. **(D)** Concentration-response curves for K_Ca_2.1 (in dark purple), K_Ca_2.2 (teal), and K_Ca_2.3 (red). Data points are mean ± SD from five independent measurements per concentration. **(E)** K_Ca_3.1 currents (in black) are insensitive to 5 µM apamin (red) but are potently blocked by 1 µM TRAM-34 (blue). **(F)** K_Ca_1.1 currents evoked by depolarization steps to +80 mV (in black) are insensitive to 5 µM of apamin (red).

**FIGURE 3:**
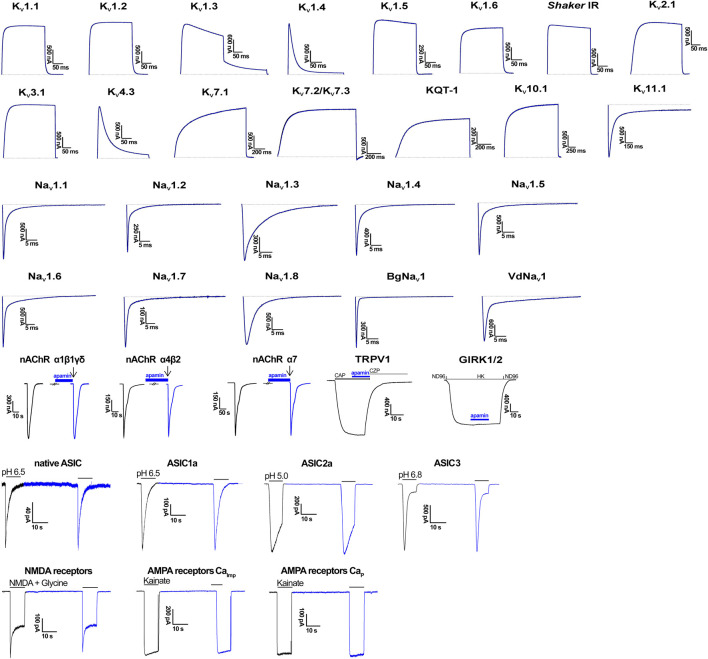
Electrophysiological profiling of apamin. Shown are representative traces of currents through the corresponding ion channels in control (gray) and after application of 5 μM toxin (blue). In the case of nAChR, TRPV1, and GIRK1/2, blue bars indicate apamin application. In nAChR, arrows indicate agonist (ACh) application. In TRPV1, the open bar shows agonist (capsaicin, CAP) application, and gray bar, antagonist (capsazepine, CZP) application. In GIRK1/2, presentation of different bath solutions is shown as line segments. In ASIC traces, application of the activating pH is shown with gray bars. And in GluR traces, gray bars depict the application of agonists. Ca_Imp_, Ca^2+^-impermeable; Ca_P_, Ca^2+^-permeable.

We estimated the activity of apamin against five K_Ca_, one K_ir_, 15 K_V_, 10 Na_V_, three ASIC, and one TRP, as well as three nAChR (Table 1; Figures 2, 3). The inhibitory effect was detected only for the three isoforms of K_Ca_2 (SK2 or SK_Ca_) channels (Figures 2A–C). We constructed concentration-response curves for the susceptible channels (Figure 2D) and confirmed that apamin displays the expected nanomolar and subnanomolar affinity to these channels. The IC_50_ values were 4.1 nM, 87.7 pM, and 2.3 nM, respectively for K_Ca_2.1, K_Ca_2.2, and K_Ca_2.3 (Table 1). Apamin had no effect on the intermediate-conductance K_Ca_3.1 (IK) or large-conductance K_Ca_1.1 (BK) currents at 5 μM (Figures 2E,F, respectively).

**TABLE 1:** Apamin potency against tested ion channels. K_Ca_ channels were expressed in HEK293 cells (K_Ca_2.3, in COS7 cells); K_V_, K_ir_, Na_V_, TRP, and nAChR were expressed in *X. laevis* oocytes; homomeric ASIC1a, ASIC2a, and ASIC3 were expressed in CHO cells; native NMDA and Ca^2+^-impermeable AMPA receptors were investigated in hippocampal CA1 pyramidal cells; native Ca^2+^-permeable AMPA receptors were studied in giant cholinergic interneurons of the striatum; and native ASIC1a/2 heteromers were investigated in hippocampal interneurons of the *lacunosum-moleculare* and *radiatum* layers of the CA1 region.


IC_50_ with 95% Confidence Interval						
Ca^2+^-activated K^+^ Channels (K_Ca_)						
K_Ca_1.1	K_Ca_2.1	K_Ca_2.2	K_Ca_2.3	K_Ca_3.1		
N.E. [1.02 ± 0.07 (*n* = 5)]	4.1 nM	87.7 pM	2.3 nM	N.E. [0.99 ± 0.05 (n = 5)]		
	95% CI 3.3–5.0 nM	95% CI 74.2–103.3 pM	95% CI 1.8–2.9 nM			
Voltage-gated K^+^ channels (K_V_)						
K_V_1.1	K_V_1.2	K_V_1.3	K_V_1.4	K_V_1.5	K_V_1.6	K_V_2.1
N.E. [0.97 ± 0.02 (*n* = 5)]	N.E. [0.99 ± 0.03 (*n* = 5)]	N.E. [1.03 ± 0.04 (*n* = 5)]	N.E. [0.99 ± 0.02 (*n* = 5)]	N.E. [0.95 ± 0.04 (*n* = 5)]	N.E. [1.02 ± 0.01 (*n* = 5)]	N.E. [1.05 ± 0.03 (*n* = 6)]
K_V_3.1	K_V_4.3	K_V_7.1	K_V_7.2/7.3	K_V_10.1	K_V_11.1	
N.E. [0.99 ± 0.02 (*n* = 5)]	N.E. [0.97 ± 0.03 (*n* = 5)]	N.E. [0.93 ± 0.05 (*n* = 5)]	N.E. [1.00 ± 0.01 (*n* = 5)]	N.E. [1.02 ± 0.04 (*n* = 6)]	N.E. [1.07 ± 0.06 (*n* = 5)]	
*Shaker*-IR	KQT-1					
N.E. [0.98 ± 0.04 (*n* = 5)]	N.E. [1.01 ± 0.02 (*n* = 5)]					
Inwardly rectifying K^+^ channels (K_ir_)						
K_ir_3.1/3.2						
N.E. [1.02 ± 0.04 (*n* = 6)]						
Voltage-gated Na^+^ channels (Na_V_)						
Na_V_1.1	Na_V_1.2	Na_V_1.3	Na_V_1.4	Na_V_1.5	Na_V_1.6	Na_V_1.7
N.E. [0.93 ± 0.04 (*n* = 5)]	N.E. [0.97 ± 0.02 (*n* = 6)]	N.E. [0.98 ± 0.06 (*n* = 5)]	N.E. [1.06 ± 0.08 (*n* = 5)]	N.E. [1.00 ± 0.03 (*n* = 5)]	N.E. [1.09 ± 0.06 (*n* = 6)]	N.E. [0.93 ± 0.08 (*n* = 5)]
Na_V_1.8	BgNa_V_1	VdNa_V_1				
N.E. [0.95 ± 0.04 (*n* = 5)]	N.E. [0.96 ± 0.06 (*n* = 5)]	N.E. [0.97 ± 0.01 (*n* = 5)]				
Transient receptor potential channels (TRP)						
TRPV1						
N.E. [1.20 ± 0.09 (*n* = 5)]						
Nicotinic acetylcholine receptors (nAChR)						
α1β1γδ	α4β2	α7				
N.E. [1.06 ± 0.03 (*n* = 6)]	N.E. [0.95 ± 0.07 (*n* = 6)]	N.E. [1.10 ± 0.08 (*n* = 6)]				
Acid-sensing ion channels (ASIC)						
ASIC1a/2 native	ASIC1a	ASIC2a	ASIC3			
N.E. [0.99 ± 0.12 (*n* = 10)]	N.E. [0.99 ± 0.03 (*n* = 6)]	N.E. [1.01 ± 0.03 (*n* = 7)]	N.E. [0.99 ± 0.02 (*n* = 7)]			
Glutamate receptors (GluR)						
AMPA (Ca^2+^-impermeable)	AMPA (Ca^2+^-permeable)	NMDA				
N.E. [0.98 ± 0.03 (*n* = 7)]	N.E. [1.01 ± 0.02 (*n* = 6)]	N.E. [0.96 ± 0.07 (*n* = 14)]				

N.E., no effect at 5 μM concentration. The current ratio (I_apamin_/I_control_) with an indication of SD, and of the *n* is displayed in the square brackets.

The effects of apamin on native iGluR (Ca^2+^-permeable and Ca^2+^-impermeable AMPA receptors as well as NMDA receptors) and ASIC were studied in isolated neurons of rat brain. Extracellular application of apamin alone did not produce noticeable effects on the holding currents. We compared the whole-cell currents through the receptors in control and in the presence of apamin. At a concentration of 5 µM, it produced no effects on the three types of iGluR and ASIC. In the case of NMDA receptors we noticed that the current in the presence of apamin tended to be slightly lower than in control (in some cells up to ≈15%), although this difference was not statistically significant (I_apamin_/I_control_ = 0.96 ± 0.07, *p* > 0.05, *n* = 14; Supplementary Figure S2).

## 4 Discussion

### 4.1 Apamin’s 3D structure

Apamin was investigated several times, by different groups, using both crystallography and NMR spectroscopy. To our surprise, no atomic coordinates were deposited in the PDB, limiting the utility of those studies to further progress. We, therefore, performed a solution NMR study, solved our own structure (Figures 1C,D), and deposited it in the PDB (accession code: 7OXF).

At least four papers reported the spatial structure of apamin. The first study used NMR and was published by Bystrov and coworkers in 1980 (Bystrov et al., 1980). However, the reported structure appears erroneous due to the wrong positioning of the secondary structure elements (for instance, the ɑ-helix was proposed to be formed in the region 6–13, which is incorrect). Two later NMR studies were published in 1983 and 1988 (Wemmer and Kallenbach, 1983; Pease and Wemmer, 1988) and reported a conformation that seems almost correct, with the ɑ-helix in the region 9–17, and a β-turn formed by residues 2–5. Finally, the most recent work by Aumelas and coworkers (Le-Nguyen et al., 2007) reported an NMR structure of apamin and an X-ray structure of its analog. The NMR structure from that study is in perfect agreement with the apamin conformation observed in our work. The X-ray structure, on the other hand, presents a dimer and differs in the conformation of the N-terminus, the length of the ɑ-helical segment (9–18 in the case of X-ray and 9–16 in NMR), and the dihedral angles of the disulfide bonds. These differences are apparently due to crystal packing constraints.

### 4.2 Apamin shows no antimicrobial effects

Honeybee *Apis mellifera* venom is well known to display antibacterial and antifungal effects, which are mostly due to its major component melittin, one of the best-studied cytolytic peptides (Habermann, 1972; Raghuraman and Chattopadhyay, 2007). Expectations of similar activity in apamin are reasonable because animals usually produce several similar toxins or even “libraries” of toxins, which is the case for both neurotoxic and cytolytic components (Vassilevski et al., 2009). However, apamin testing against Gram-positive and Gram-negative bacteria revealed no effect up to a very high concentration of 50 μM. At the same time, melittin shows prominent antimicrobial effects at low micromolar concentrations, and since its content in bee venom is ≈ 20 times higher, the chances that apamin shares a similar mode of action are very low.

### 4.3 Apamin’s revisited pharmacology

Small-conductance Ca^2+^-activated K^+^ channels (K_Ca_2, SK, or SK_Ca_) are a group of three α-subunit isoforms (K_Ca_2.1–2.3) that can form mature channels of either homo- or heterotetrameric structure (Köhler et al., 1996; Strassmaier et al., 2005). Similarly to many other K^+^ channels, each SK α-subunit contains six transmembrane segments (S1–S6, with S5 and S6 contributing to the pore domain). Unlike K_V_ channels or large-conductance Ca^2+^-activated K^+^ channels (BK or K_Ca_1.1), the gating of SK channels is insensitive to transmembrane voltage. K_Ca_2 channels have no Ca^2+^ binding sites in their α-subunit, but they form a stable complex with calmodulin that acts as their Ca^2+^ sensor (Xia et al., 1998). All three isoforms of K_Ca_2 are widely expressed in the central nervous system (Stocker and Pedarzani, 2000; Chen et al., 2004) and found in sensory neurons and the heart (Köhler et al., 1996; Xu et al., 2003; Tuteja et al., 2005; Skibsbye et al., 2014); K_Ca_2.2 is also important in the liver (Feranchak et al., 2004); and K_Ca_2.3 is found in many tissues (Herrera et al., 2003; Tamarina et al., 2003; Chen et al., 2004). According to the Mouse Brain Atlas (http://www.mousebrain.org) (Zeisel et al., 2018), K_Ca_2.1 is highly expressed in neurons of the cerebral cortex, midbrain red nucleus, hindbrain, and sensory neurons; K_Ca_2.2 in the cerebral cortex, thalamus, hippocampus, spinal cord, midbrain, hindbrain, erector muscle, and sensory neurons, as well as glial cells; and K_Ca_2.3 in the diencephalon, nuclei of cranial nerves, medulla, thalamus, hypothalamus, ventral midbrain, hindbrain, cerebellum, enteric and erector muscle neurons, as well as enteric fibroblasts.

The number of known polypeptide or peptide toxins acting on K_Ca_2 with high (nanomolar) affinity is limited. According to the Kalium database (https://kaliumdb.org) (Kuzmenkov et al., 2016; Tabakmakher et al., 2019), six of these substances were identified in scorpion venom (AmP05, maurotoxin, Pi-1, scyllatoxin, tamapin, and Ts9). Scyllatoxin (α-KTx 5.1) from *Leiurus quinquestriatus hebraeus* (Shakkottai et al., 2001) and tamapin (α-KTx 5.4) from *Mesobuthus tamulus* (Pedarzani et al., 2002), in particular, show high potency against K_Ca_2.2 with IC_50_ values in the subnanomolar range. Interestingly, apamin shares a similar sequence motif RXCQ with several scorpion toxins that inhibit K_Ca_ channels: X = R in apamin and AmP05, M in scyllatoxin, and P in Pi-1. In all these toxins the RXCQ motif is found in an α-helix. Replacement of “X” in the RXCQ motif of scyllatoxin with a positively charged residue (e.g., lysine or 2,4-diaminobutyric acid) resulted in derivatives with enhanced selectivity for K_Ca_2.2 over K_Ca_2.3 (Sabatier et al., 1993, 1994; Shakkottai et al., 2001). The importance and generality of this motif will be clarified in the future.

Structure-activity studies showed that one of the two adjacent arginine residues (Arg13 and Arg14) and Gln17 of apamin are key determinants of its activity (Vincent et al., 1975; Sandberg, 1979; Labbé-Jullié et al., 1991). Mutagenesis suggested that one of these Arg residues interacts with Asp341 (S5–P region) of K_Ca_2.2 channel pore region, whereas Gln17 interacts with Asn368 (P–S6; numbering according to rK_Ca_2.2, UniProt accession number: P70604) (Ishii et al., 1997). Two additional positions in the pore region were subsequently proposed to be involved in apamin sensitivity [His337 or His485 of K_Ca_2.2 and K_Ca_2.3 (Q9UGI6), respectively, and Asn345 of K_Ca_2.2; all in S5–P] (Lamy et al., 2010). Those studies advocated in favor of apamin acting like a pore blocker. On the other hand, some investigations reported that the S3–S4 extracellular loop may be an essential molecular determinant of apamin sensitivity suggesting an allosteric mode of action and not direct pore blockage. Thus, a point mutation (Thr216Ser) significantly influences K_Ca_2.1 (Q92952) sensitivity towards apamin (Nolting et al., 2007). In addition, the three-amino-acid motif in the S3–S4 loop of K_Ca_2 channels (^216^TYA^218^ in hK_Ca_2.1, ^244^SYA^246^ in hK_Ca_2.2, and ^393^SYT^395^ in hK_Ca_2.3) was implicated in forming the binding interface for apamin (Weatherall et al., 2011). In the absence of a solved 3D structure of a K_Ca_2 channel, it is difficult to predict the details of molecular interactions with apamin. One tempting possibility is that the long S3–S4 loop protrudes to the pore domain and together they form a common binding site for apamin.

It is apamin from the honeybee venom (Habermann and Reiz, 1965; Habermann, 1972) that is the most prominent peptide ligand of K_Ca_2. Indeed, since the early 1980s apamin has been used as the main pharmacological agent to distinguish K_Ca_2 channels from other K^+^ channels (Burgess et al., 1981; Romey and Lazdunski, 1984; Pennefather et al., 1985). Subsequent studies on cloned K_Ca_2 confirmed that these three channel isoforms are the molecular targets of apamin (Köhler et al., 1996; Shah and Haylett, 2000; Strøbaek et al., 2000; Grunnet et al., 2001a). However, later studies also claimed some off-target activities of apamin. One series of publications reported inhibition of Ca^2+^ and Na^+^ channels in embryonic heart tissues (Bkaily et al., 1985, 1991, 1992). And another study pointed to K_V_1.3 as a target (Voos et al., 2017). Our extensive electrophysiological measurements disagree with or directly disprove these claims. Apamin does not present any activity on neither of the expressed Na^+^ channels nor K_V_1.3.

## Data Availability

The original contributions presented in the study are included in the article/Supplementary Material, further inquiries can be directed to the corresponding author. NMR chemical shifts, experimental restraints, and the spatial structure of apamin were deposited to the BMRB (accession code 34641) and PDB databases (7OXF).
