# Supplementary material for: Apamin structure and pharmacology revisited
Source: Front Pharmacol. 2022 Sep 16;13:977440. doi: 10.3389/fphar.2022.977440 (PMC9523135; doi:10.3389/fphar.2022.977440)
Supplement: Supplementary file 1 [file DataSheet1.docx]

Supplementary Materials


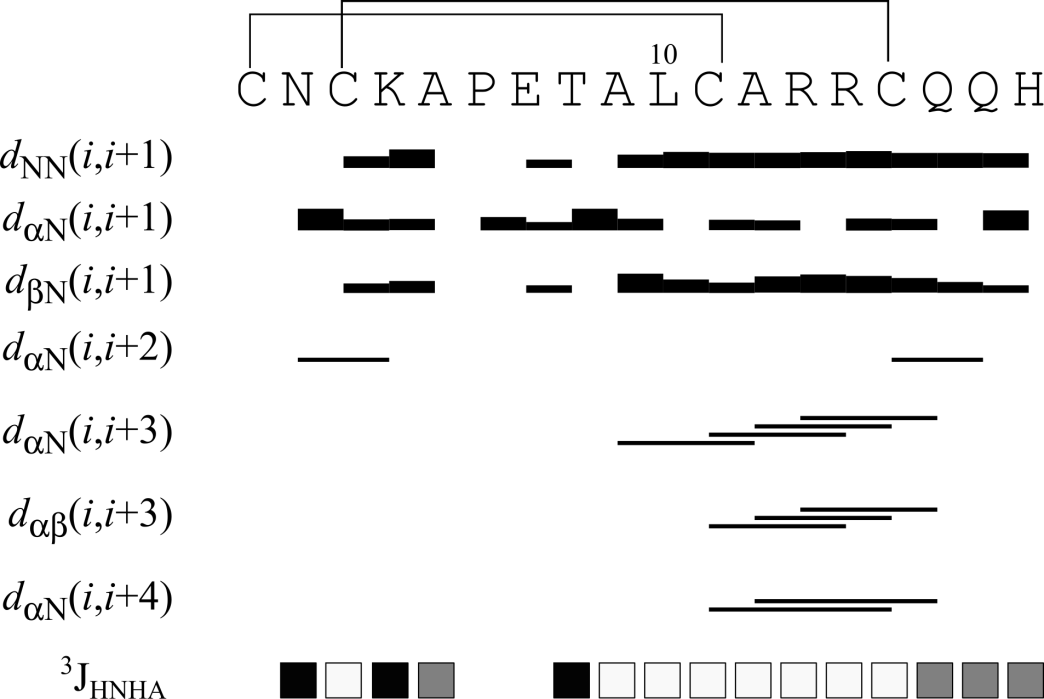


**Figure S1.** Experimental data used for the structure determination of apamin. The following parameters are listed top down: protein sequence with S-S-bonds, NOE connectivities (d_ij_), and ^3^J_HNHA_ couplings. Widths of the bars represent the relative intensity of cross-peaks in NOESY spectra. Squares have three colors according to the value of J-coupling: black (>8 Hz), gray (6–8 Hz), and white (<6 Hz).

**Figure S2**. Absolute NMDA current amplitudes in control and in the presence of 5µM apamin.

Table S1. Statistics for the 10 best NMR structures of apamin.

| Parameter | Value |
| --- | --- |
| **Distance and angle restraints** | |
| Total NOEs | 142 |
| Intraresidual | 54 |
| Interresidual | 88 |
| Sequential (\|i −j\|=1) | 45 |
| Medium range (1*<*\|i −j\|≤4) | 35 |
| Long range (\|i −j\|*>*4) | 8 |
| S-S bond restraints (upper/lower) | 6/6 |
| Angles: |  |
| φ | 15 |
| χ^1^ | 5 |
| Total restraints per residue | 9.7 |
| **Statistics for the calculated set of structures** | |
| CYANA target function (Å^2^) | 0.69±0.06 |
| Restraint violations: |  |
| Distance (*>*0.3 Å) | 0 |
| Angle (*>*5°) | 1 |
| RMSD (Å) (residues 2–17) |  |
| Backbone | 0.24±0.16 |
| All heavy atoms | 1.24±0.29 |
| **Ramachandran analysis** | |
| % residues in most favored regions* | 86.7 |
| % residues in additional allowed regions | 13.3 |
| % residues in generally allowed regions | 0 |
| % residues in disallowed regions | 0 |
